# A Single Amino Acid Residue R144 of SNX16 Affects Its Ability to Inhibit the Replication of Influenza A Virus

**DOI:** 10.3390/v14040825

**Published:** 2022-04-15

**Authors:** Wenjun Shi, Li Jiang, Miaomiao Ye, Bo Wang, Yu Chang, Zhibo Shan, Xuyuan Wang, Yuzhen Hu, Hualan Chen, Chengjun Li

**Affiliations:** 1 College of Veterinary Medicine, Gansu Agricultural University, Lanzhou 730070, China; duoyanji@126.com (W.S.); sankg21@163.com (X.W.); 2 State Key Laboratory of Veterinary Biotechnology, Harbin Veterinary Research Institute, Chinese Academy of Agricultural Sciences, Harbin 150069, China; jiangli@caas.cn (L.J.); yemiaomiao1996@163.com (M.Y.); wangbocaas@163.com (B.W.); changyu204@sina.com (Y.C.); szb1225@163.com (Z.S.); hyzde163yx@163.com (Y.H.)

**Keywords:** SNX16, R144 residue, influenza A virus, replication

## Abstract

Influenza A virus (IAV) is an important zoonotic pathogen, posing a severe burden for the health of both animals and humans. Many host factors are involved in the life cycle of IAV to regulate its replication. Herein, we identified sorting nexin-16 (SNX16) as a new host factor that negatively modulates the replication of IAV. When transiently overexpressed in cells, SNX16 appears to be expressed as two obvious bands. Mutagenesis analysis indicated that the amino acid residue R144 of SNX16 was responsible for its two-band expression phenotype. We found that the R144A mutation of SNX16 changed its cellular distribution in A549 cells and partially weakened the inhibitory effect of SNX16 on IAV replication. Further investigation revealed that SNX16 could negatively regulate the early stage of the replication cycle of IAV. Taken together, our results demonstrated that SNX16 is a novel restriction host factor for the replication of IAV by engaging in the early stage of IAV life cycle, and a single amino acid residue at position 144 plays an important role in the cellular distribution and anti-influenza function of SNX16.

## 1. Introduction

Influenza virus belongs to the Orthomyxoviridae family, which is classified into types A, B, C, and D, based on the antigenic distinction of its nucleoprotein (NP) and matrix protein (M) [1,2,3]. Among which, influenza A virus (IAV) is the most important pathogen with the strongest contagiousness and harmfulness to the poultry industry and public health [4]. The genome of IAV is composed of eight single stranded negative sense RNA segments. IAV is divided into 18 HA subtypes and 11 NA subtypes on the basis of the two surface glycoproteins of the virus, hemagglutinin (HA) and neuraminidase (NA) [5]. IAV continuously evolves in nature due to two forms of variation, mutation and reassortment, which are caused by the RNA character and segmented feature of its genome. The endless evolution of IAV leads to variation of the viral antigenicity, including frequent antigenic drift and occasional antigenic shift [6,7], which pose a great threat to the prevention and control of IAV. 

The eight RNA segments of the IAV genome encode 10 essential proteins: polymerase basic proteins 1 (PB1) and 2 (PB2), polymerase acidic protein (PA), HA, NP, NA, matrix proteins 1 (M1) and 2 (M2), and non-structural proteins 1 (NS1) and 2 (NS2). Among them, NP wraps the viral RNA and is bound with its ends by PB2, PB1, and PA to form the viral ribonucleoprotein (vRNP) complex [8,9]. vRNP complex is responsible for the transcription and replication of the viral genome [10,11]. In order to deliver vRNP complex into the nucleus of infected cells, IAV takes advantage of endocytosis and the cytoplasmic transport mechanisms of host cells to accomplish the invasion process. Firstly, IAV recognizes and binds to sialic acid receptors on the surface of host cells. After binding, IAV enters cells through clathrin-mediated endocytosis or macropinocytosis pathway [12,13]. IAV is then transported to the early endosome via cytoplasmic transport. At this point, the PH value drops to 5~5.5, and such a low PH environment induces irreversible changes in the conformation of HA protein, resulting in activation of its membrane fusion activity [14]. As a result, the membrane fusion between IAV and the late endosome membrane occurs, and the viral capsid protein is released into the cytoplasm, which is called uncoating. In the cytoplasm, M1 disassociates from the vRNP complex, which is required for vRNP to enter into the nucleus [15]. The vRNP complex then translocates into the nucleus via the classical nuclear import pathway, followed by the initiation of transcription and replication of the viral genome [16]. 

The most effective measure to prevent and control IAV is vaccination [17,18,19]. However, the frequency of the antigenic variation of IAV urges updates of vaccinations, which makes vaccination lag slightly behind prevention requirements. On the other hand, antiviral drugs play an important role in the treatment of influenza infections. So far, three generations of anti-influenza drugs had been developed and applied in treating IAV infection, including the M2 ion channel inhibitors (e.g., amantadine), the NA inhibitors (e.g., oseltamivir), and polymerase inhibitors (e.g., baloxavir). The common feature of these anti-influenza drugs is that they all target the viral proteins of IAV. Although these drugs have played an important role in the treatment of IAV infections, their effectiveness is also compromised by the appearance of drug resistant mutations during the drug usage [20,21,22]. To develop novel anti-influenza drugs, host factors participating in the life cycle of IAV could be selected as potential targets. Hence, exploring the mechanism of how host factors participate in the life cycle of IAV is of significant importance.

SNX16 consists of 343 amino acids with a coiled-coil (CC) domain next to the C-terminus of the PX domain, which mediates the association with the membrane by interacting with the phospholipid phosphatidylinositol 3-phosphate [23]. The CC domain of SNX16 plays an important role in its higher-order assembly and the regulation of membrane tubulation [24]. SNX16 may function in the trafficking of proteins between the endosomal compartments. It is reported that SNX16 is distributed to the early endosome, late endosome/lysosome, recycling endosome, and cell cortex [23,25,26,27,28], and that subcellular distribution of SNX16 at the cell cortex reveals the presence of an SNX23- and microtubule-dependent cargo transport pathway, in which SNX16 negatively regulates cell migration and tumorigenesis [28]. It is reported that in CRC tissues, the expression of SNX16 is significantly upregulated, and upregulated SNX16 interacts with translation elongation factor 1A2 (eEF1A2) to inhibit the degradation and ubiquitination of eEF1A2, thereby activating c-Myc signaling to promote colorectal tumorigenesis [29]. By immunoprecipitation and mass spectrometry screening, we identified that host factor eEF1A2 could negatively regulate the replication of IAV (data not shown). However, whether SNX16 plays a role in the replication of IAV remains unclear.

## 2. Materials and Methods

### 2.1. Cells and Virus

Human lung carcinoma cells (A549, ATCC CCL-185) and human embryonic kidney cells (HEK293T, ATCC CRL-3216) were cultured in F12K (Life Technologies, Grand Island, NY, USA) with 10% fetal bovine serum (FBS, Sigma-Aldrich, Saint Louis, MO, USA); Madin–Darby canine kidney (MDCK) cells were cultured in DMEM, supplemented with 5% newborn calf serum (Sigma-Aldrich). All media were supplemented with 100 U/mL penicillin and 100 µg/mL streptomycin (Life Technologies). Cells were incubated with 5% CO_2_ at 37 °C. 

The A/WSN/33 (WSN, H1N1) strain of influenza A virus was grown in MDCK cells, cultured in MEM supplemented with 0.3% bovine serum albumin (BSA, Sigma-Aldrich) and 0.5 µg/mL l-1-tosylamide-2-phenylmethyl chloromethyl ketone (TPCK)-treated trypsin (Worthington, Lakewood, NJ, USA). 

### 2.2. Plasmids

The SNX16 gene was amplified from cDNAs of A549 cells by reverse transcription-PCR amplification of total cellular mRNA with Superscript III reverse transcriptase (Invitrogen, Carlsbad, CA, USA). The amplified SNX16 gene was subsequently cloned into the mammalian expression vector pCAGGS, with or without a Flag, V5, or HA-tag at the N-terminus. The R144A mutation was introduced into SNX16 by the PCR approach, and the mutagenized SNX16 gene was cloned into the pCAGGS vector, with or without a Flag tag at the N-terminus. All constructs were validated by sequencing.

### 2.3. Antibodies

Mouse anti-NP monoclonal antibody (mAb) was prepared in our laboratory [30]. The following primary antibodies were obtained from commercial resources: rabbit anti-V5 polyclonal antibody (pAb) (AB3792, Merck Millipore, Billerica, MA, USA); rabbit anti-GAPDH pAb (10494-1-AP) and rabbit anti-HA tag pAb (51064-2-AP, Proteintech, Nanjing, China); mouse anti-Flag mAb (F3165) and rabbit anti-Flag pAb (F7425) from Sigma-Aldrich; and mouse anti-actin mAb (sc-47778) and mouse anti-SNX16 mAb (sc-271260, Santa Cruz, Dallas, TX, USA). The secondary antibodies used in Western blotting were Dylight 800 goat anti-rabbit IgG (RS23920) and Dylight 800 goat anti-mouse IgG (RS23910), purchased from Immunoway (Newark, DE, USA); the secondary antibodies used in confocal microscopy were Alexa Fluor 488 goat anti-rabbit IgG (H + L) (A11034) and Alexa Fluor 633 goat anti-mouse IgG (H + L) (A21052), obtained from Invitrogen.

### 2.4. siRNA Knockdown, Plasmid Transfection and Virus Infection

Scrambled siRNA or siRNA targeting SNX16 (5′-CCAGUUAGAAGACUCAAAUTT-3′; 5′-AUUUGAGUCUUCUAACUGGTT-3′) was transfected into A549 cells by using Lipofectamine RNAi MAX (Invitrogen). At 48 h post transfection, the knockdown efficiency of SNX16 was determined by means of RT-qPCR.

Empty vector or plasmid expressing Flag-tagged SNX16 was transfected into A549 cells by using Lipofectamine 2000 (Invitrogen). At 48 h post transfection, the expression of SNX16 was detected by means of Western blotting. 

siRNA-treated and plasmid-transfected A549 cells, as indicated above, were infected with WSN (H1N1) virus at an MOI of 0.01. Supernatants were collected at 24 and 48 h post-infection (p.i.) and virus titers were determined by means of plaque assays on MDCK cells.

### 2.5. qRT-PCR Assays

qRT-PCR assays were performed by using SYBR Premix Ex Taq II (Tli RNase H Plus; TaKaRa, Kusatsu, Shiga, Japan). Relative RNA quantities were determined by using the comparative cycle threshold method, with the cellular GAPDH gene serving as the internal control. Dissociation curve analysis was performed after each assay to ensure specific detection. 

### 2.6. Western Blotting

Cells were lysed with 1 × SDS lysis buffer and boiled for 10 min. Then, the cell lysates were centrifuged at 4000 rpm for 3 min. The supernatants were subjected to SDS-PAGE and then transferred onto nitrocellulose membranes. The membranes were blocked at room temperature for 1 h with 5% skim milk, followed by incubation overnight at 4 °C with appropriately diluted primary antibodies. After three washes with PBST, the membranes were incubated with the indicated secondary antibodies at room temperature for 1 h and washed with PBST three times. Finally, the membranes were visualized by using an Odyssey CLX infrared imaging system (Li-Cor BioScience, Lincoln, NE, USA).

### 2.7. Cell Viability Assay

Cell viability was determined as previously described [31,32]. Briefly, cells seeded in opaque-walled 96-well plates were treated as indicated and were subsequently incubated with 100 µL of CellTiter-Glo reagent at room temperature for 10 min on a shaker. The luminescence was measured by a GloMax 96 Microplate Luminometer (Promega, Madison, WI, USA).

### 2.8. Confocal Microscopy

HEK293T cells were transfected with the indicated plasmids by using Lipofectamine LTX (Invitrogen), and A549 cells were transfected with the indicated siRNA by using Lipofectamine RNAi MAX. At 48 h post transfection, cells were infected with WSN (H1N1) virus at an MOI of 5. At 2 h p.i., cells were fixed with 4% paraformaldehyde (PFA) for 1 h, permeabilized with 0.5% Triton X-100 in PBS for 15 min, and blocked with 5% BSA in PBS for 1 h. The cells were then incubated with a rabbit anti-Flag pAb (1:100) and/or a mouse anti-NP mAb (1:100), at 4 °C overnight, followed by incubation with secondary antibodies (Alexa Fluor 488 goat anti-rabbit IgG (H + L) and/or Alexa Fluor 633 goat anti-mouse IgG (H + L)) for 1 h. The nuclei of the cells were stained with DAPI (4′,6-diamidino-2-phenylindole, Thermo Fisher Scientific, Waltham, MA, USA) for 5 min. Images were acquired by using an LSM 980 confocal microscope (Zeiss, Oberkochen, Germany).

### 2.9. Plaque Assay

Virus titers were determined by means of plaque assays on MDCK cells. Briefly, MDCK cells grown in 12-well plates until 95% confluency, were infected with WSN (H1N1) virus at an MOI of 0.01. After an incubation of 1 h at 37 °C, the inoculum was removed, and the cells were washed with 1 × MEM and overlaid with 1% SeaPlaque agarose (Lonza, Rockland, ME, USA) in 1 × MEM (0.3% BSA, 0.5 µg/mL TPCK-treated trypsin) until solidification at room temperature. Then, the cells were incubated for 48 h at 37 °C, and the plaques were counted.

## 3. Results

### 3.1. Overexpression of SNX16 Inhibits the Replication of IAV

**Figure 1:**
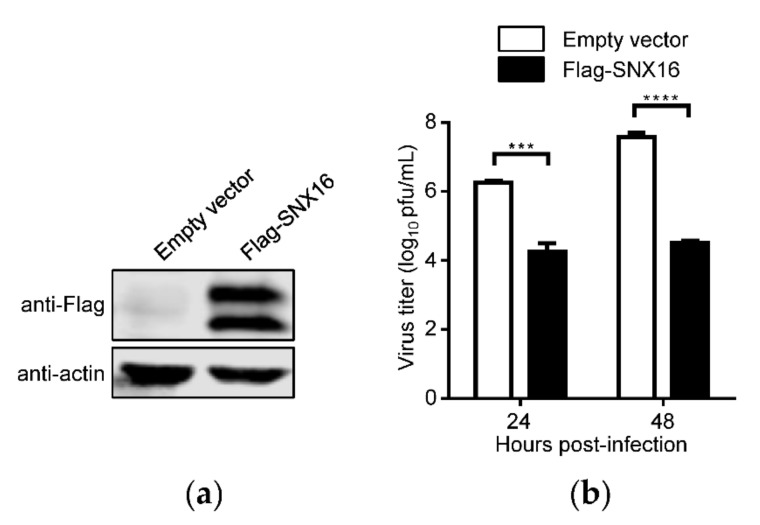
Overexpression of SNX16 inhibits the replication of IAV. (**a**) A549 cells were transfected with empty vector or plasmids expressing Flag-SNX16. The expression of SNX16 was subjected to Western blotting with a mouse anti-Flag mAb. (**b**) The transfected cells as in (**a**) were infected with WSN (H1N1) virus at an MOI of 0.01. Supernatants were collected at the indicated time points to determine the virus titers by plaque assay (*n* = 3). ***, *p <* 0.001; ****, *p <* 0.0001. Data are representative of three independent experiments.

In a preliminary immunoprecipitation and mass spectrometry screening, we identified eEF1A2 as a potential restricting factor for IAV replication (data not shown). To investigate whether SNX16, an interacting partner of eEF1A2 [29], is related to the replication of IAV, we determined the effect of SNX16 overexpression on the growth titers of IAV in A549 cells. As shown in Figure 1a, the expression of Flag-tagged SNX16 was well detected by using a mouse anti-Flag mAb. Interestingly, we found that two obvious bands of Flag-SNX16 were expressed compared with the empty vector (Figure 1a). The empty vector-transfected cells and SNX16-overexpressing cells were infected with WSN (H1N1) virus at an MOI of 0.01. We found that the transient overexpression of SNX16 in A549 cells led to a 100- and 1156-fold reduction of the growth titer of WSN (H1N1) virus at 24 and 48 h p.i., respectively (Figure 1b), indicating that the overexpression of SNX16 significantly inhibits the replication of IAV. 

### 3.2. Downregulation of SNX16 Expression Promotes the Replication of IAV

**Figure 2:**
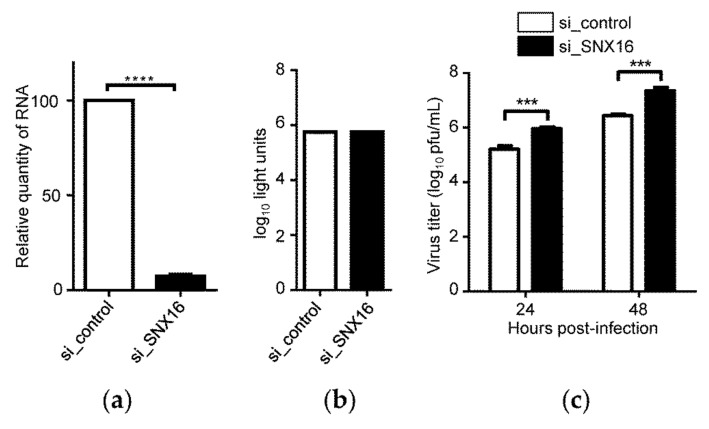
Downregulation of SNX16 expression promotes the replication of IAV. (**a**) A549 cells were transfected with siRNA targeting SNX16 or with scrambled siRNA for 48 h and the expression of SNX16 was detected by RT-qPCR (*n* = 3), ****, *p <* 0.0001. (**b**) Viability of A549 cells treated with SNX16-specific or scrambled siRNA was determined by using a CellTiter-Glo assay (*n* = 3). (**c**) A549 cells treated with siRNA for 48 h were infected with WSN (H1N1) virus (MOI = 0.01). Supernatants were collected at 24 and 48 h p.i., and virus titers were determined by plaque assays on MDCK cells (*n* = 3). ***, *p <* 0.001. Data are representative of three independent experiments.

To further validate whether SNX16 plays a role in the replication of IAV, A549 cells were transfected with siRNA targeting SNX16 to downregulate its expression. We found that the transfection with SNX16-specific siRNA significantly reduces the expression of SNX16 by quantitative reverse transcription PCR (RT-qPCR) (Figure 2a) and siRNA treatment caused no effect on cell viability (Figure 2b). The siRNA-treated cells were infected with WSN (H1N1) virus (MOI = 0.01). As shown in Figure 2c, siRNA knockdown of SNX16 expression led to a 5.6- and 8.3-fold increase in the growth titer of IAV at 24 and 48 h p.i., respectively (Figure 2c), confirming that SNX16 is a restricting factor for the replication of IAV. 

### 3.3. Transient Overexpression of SNX16 Exhibits Two Obvious Bands

**Figure 3:**
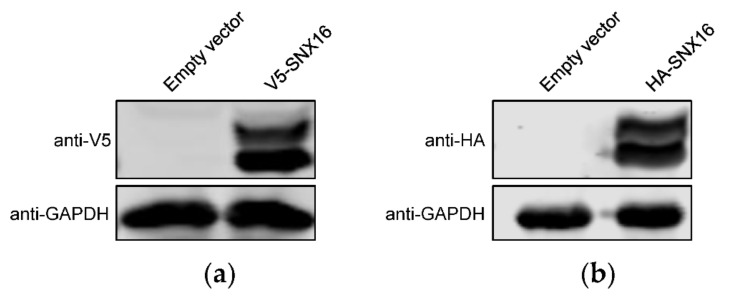
SNX16 is expressed as two obvious bands. HEK293T cells were transfected with plasmids expressing V5- (**a**) and HA-tagged (**b**) SNX16, and were subjected to Western blotting with a rabbit anti-V5 pAb and a rabbit anti-HA pAb, respectively. Data are representative of three independent experiments.

As shown in Figure 1a, the transfection of Flag-SNX16-expressing plasmids resulted in two separated bands by Western blotting. To further validate this phenomenon, we generated two additional SNX16-expressing constructs bearing an HA and V5 tag, respectively. HEK293T cells were transfected with the two constructs, followed by Western blotting to detect the expression of SNX16. As shown in Figure 3a,b, both V5-SNX16 and HA-SNX16 were expressed as two obvious bands. 

### 3.4. A Single Amino Acid Residue R144 Is Critical for the Expression and Distribution of SNX16

**Figure 4:**
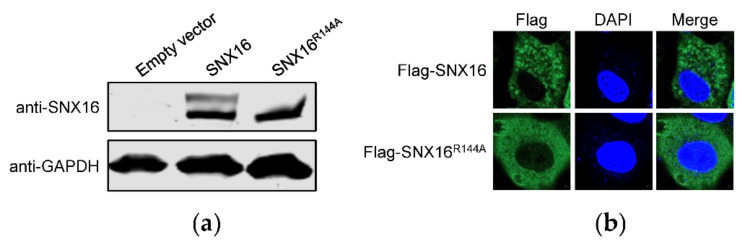
A single amino acid R144 is essential for the expression and distribution of SNX16. (**a**) A549 cells were transfected with plasmids expressing empty vector, pCAGGS-SNX16, or pCAGGS-SNX16^R144A^, and then subjected to Western blotting with a mouse anti-SNX16 mAb. (**b**) A549 cells were transfected with plasmids expressing Flag-SNX16 or Flag-SNX16^R144A^, followed by confocal microscopy with a rabbit anti-Flag pAb. Data are representative of two independent experiments.

It is reported that a single amino acid mutation R144A of SNX16 could abolish its binding to membranes enriched in phosphatidylinositol 3-phosphate [33], indicating that R144 of SNX16 plays an important role in the function of SNX16. To explore whether the expression of SNX16 as two bands is possibly related to R144, we generated an SNX16-expresssing construct containing the R144A mutation and determined its effect on the expression of SNX16. We found that the upper band of SNX16 disappeared due to the introduction of the single R144A mutation (Figure 4a), indicating that R144 plays an important role for the expression of SNX16. 

To further determine the influence of the R144A mutation of SNX16, A549 cells were transfected with plasmids expressing Flag-SNX16 and Flag-SNX16^R144A^, and were subjected to confocal microscopy to examine the expression of SNX16. We found that both Flag-SNX16 and Flag-SNX16^R144A^ were located in the cytoplasm (Figure 4b). However, the distribution status of Flag-SNX16^R144A^ is different from that of Flag-SNX16: Flag-SNX16 gathered as dot structures, whereas Flag-SNX16^R144A^ is diffusely distributed in the cytoplasm (Figure 4b). These results indicated that a single R144A mutation changed the intrinsic expression and distribution status of SNX16 in A549 cells.

### 3.5. Homology Analysis of R144 of SNX16

**Figure 5:**
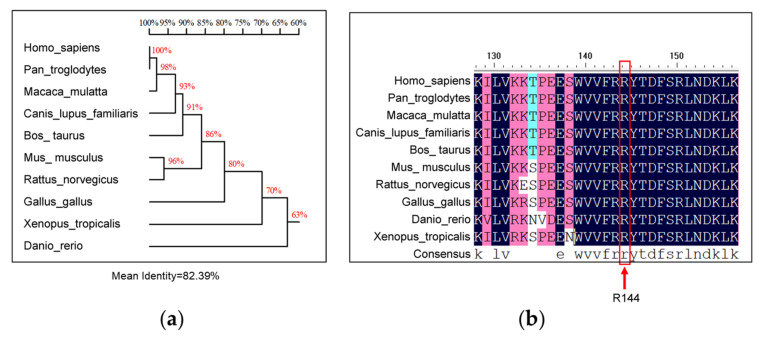
Homology analysis of residue R144 of SNX16. The amino acid sequences of SNX16 protein of different species from NCBI were aligned by using DNAMAN software to show homologies of SNX16 (**a**) and the conservativeness of residue R144 (**b**).

In order to evaluate the conservativeness of the R144 residue of SNX16 in different species, the amino acid sequences of 10 SNX16 protein sequences from different species were derived from the NCBI (https://www.ncbi.nlm.nih.gov/ accessed on 31 December 2021) and were aligned by using DNAMAN software. The results of sequence alignments showed that the sequence homologies of SNX16 ranged from 100% to 63% among different species (Figure 5a), and the residue R144 was 100% conserved among different species (Figure 5b). 

### 3.6. R144A Mutation of SNX16 Partially Attenuates Its Inhibitory Effect on IAV Replication

**Figure 6:**
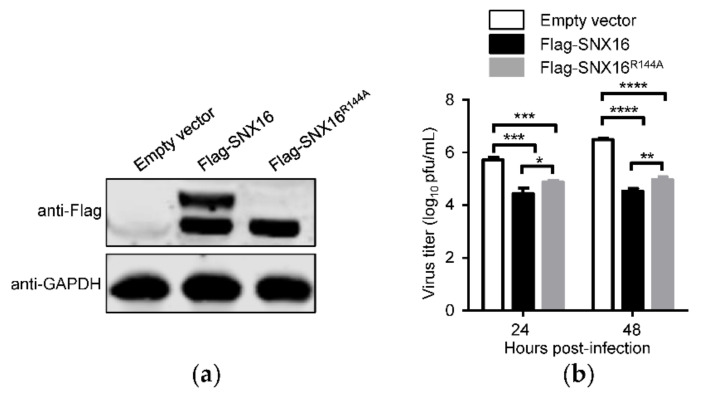
R144A mutation of SNX16 attenuates its inhibitory effect on IAV replication. (**a**) A549 cells were transfected with plasmids expressing empty vector, Flag-SNX16, and Flag-SNX16^R144A^. The expression of SNX16 was detected by Western blotting. (**b**) The transfected A549 cells, as in (**a**), were infected with WSN (H1N1) virus at an MOI of 0.01. Supernatants were collected at the indicated time points and virus titers were evaluated by plaque assay (*n* = 3). *, *p <* 0.05; **, *p <* 0.01; ***, *p <* 0.001; ****, *p <* 0.0001. Data are representative of three independent experiments.

To determine whether the R144 residue of SNX16 is related to the replication of IAV, A549 cells were transfected with plasmids expressing empty vector, Flag-SNX16, or Flag-SNX16^R144A^, and were subjected to Western blotting to check the expression of the indicated proteins (Figure 6a). The transfected cells were infected with WSN (H1N1) virus at an MOI of 0.01 and viral growth titers were measured by plaques assays. We found that the replication of IAV was significantly inhibited by the expression of SNX16, while the introduction of the R144A mutation partially recovered the replication of IAV (Figure 6b). These results indicate that the R144 residue plays a role in the inhibitory effect of SNX16 on the replication of IAV. 

### 3.7. SNX16 Functions in the Early Stage of IAV Replication Cycle

**Figure 7:**
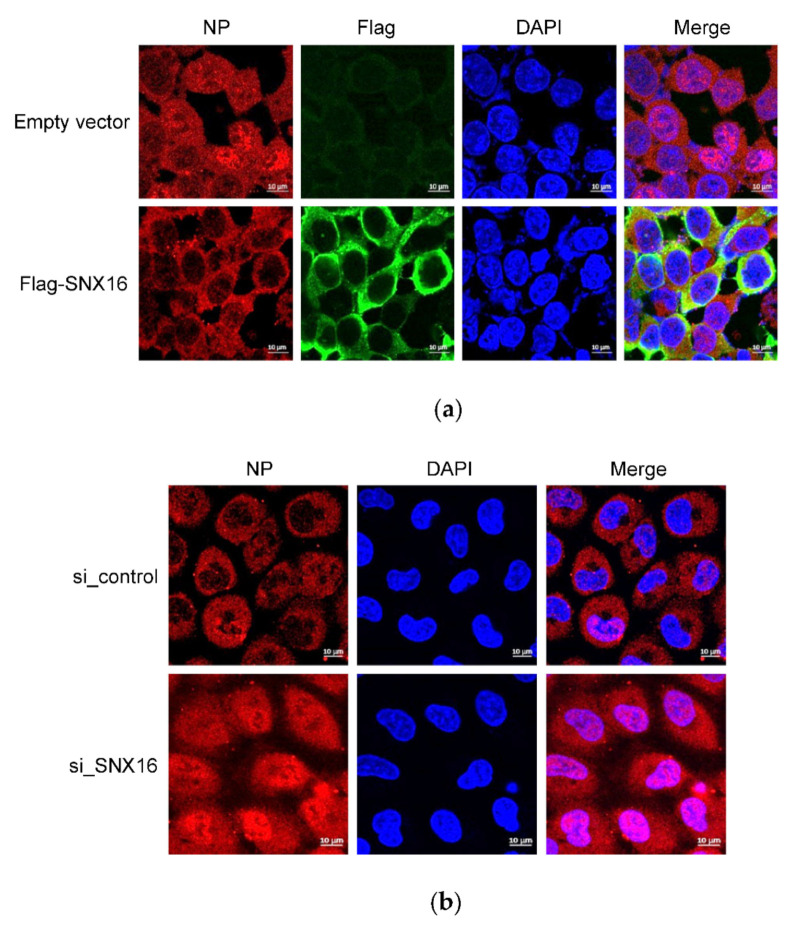
SNX16 is involved in the early stage of IAV replication cycle. (**a**) HEK293T cells were transfected for 48 h to express empty vector or Flag-SNX16, and then infected with WSN (H1N1) virus at an MOI of 5. At 2 h p.i., the infected cells were fixed and stained with a mouse anti-NP mAb and a rabbit anti-Flag pAb, followed by incubation with Alexa Fluor 633 goat anti-mouse IgG (H + L) (red) and Alexa Fluor 488 goat anti-rabbit IgG (H + L) (green). The nuclei were stained with DAPI. (**b**) A549 cells were transfected with siRNA targeting SNX16 or with scrambled siRNA for 48 h and were then infected with WSN (H1N1) virus at an MOI of 5. At 2 h p.i., the infected cells were fixed and stained with a mouse anti-NP mAb, followed by incubation with Alexa Fluor 633 goat anti-mouse IgG (H + L) (red). The nuclei were stained with DAPI. Data are representative of three independent experiments.

To gain an insight on the role of SNX16 in the life cycle of IAV, HEK293T cells were transfected to express empty vector or Flag-SNX16, followed by infection with WSN (H1N1) virus at an MOI of 5. At 2 h p.i.; the cells were subjected to confocal microscopy assay to reveal the expression of SNX16 and IAV NP. As shown in Figure 7a, NP protein was obviously accumulated in the nucleus of the control cells but was confined in the cytoplasm of SNX16-overpressing cells, indicating that SNX16 functions as a restricting factor in the early stage of the IAV life cycle. To further validate this finding, A549 cells were treated with scrambled siRNA or siRNA targeting SNX16 and were subsequently infected with WSN (H1N1) virus (MOI = 5). Confocal microscopy assay showed that at 2 h p.i., the accumulation of NP in the nucleus of SNX16-siRNA treated cells was significantly stronger than that of the control cells (Figure 7b). Together, these results indicated that SNX16 negatively regulates the early stage of the IAV life cycle. 

## 4. Discussion

IAV unceasingly poses tremendous threats to humans and animals in that it could continuously evolve in nature through mutation and reassortment to rebuild its segmented RNA genome [34,35,36,37,38]. Vaccination is an important countermeasure in the fight against IAV, especially when the vaccine seed virus can be timely updated based on the antigenicity change of the epidemic strains [7,17,18,39]. However, as IAV is prone to mutation, the prediction and selection of an antigenically matched vaccine seed virus is not always successful, thereby limiting the effectiveness of vaccination in certain human influenza seasons [40,41]. On the other hand, the use of anti-influenza drugs is another important means in dealing with the threats of IAV. Given that the use of current anti-IAV drugs is facing the challenge of drug resistant mutants [20,21,22], the employment of new strategies to develop novel anti-IAV drugs is highly desired.

The intrinsic property of IAV is host dependence, which means that the infection could not occur without the presence of a host, and the completion of the IAV life cycle relies on numerous host factors. Meanwhile, some host factors could restrict the replication of IAV at different stages of the viral life cycle. Hence, critical host factors participating in the life cycle of IAV could be potential targets for the development of novel anti-IAV drugs. To date, a large number of host factors have been identified to be involved in the life cycle of IAV. Take the host factors affecting the entry process of IAV as examples; FFAR2 and IGDCC4 are involved in the internalization process during IAV entry [42,43]; CD81 assists viral fusion by promoting the trafficking of IAV to fusion-competent endosomes and further organizes the endosomal membrane [44]; Cathepsin W is required for the escape of IAV from the late endosome to proceed to the next stage of invasion [45]; SPOPL affects the uncoating process of IAV entry through a CUL3-SPOPL E3 ubiquitin ligase complex, to ubiquitinate and degrade EPS15 at the endosomes [46]; and Itch ubiquitinates the viral M1 protein in endosomes and mediates the release of vRNP complexes from the endosomal compartments [47]. It is expected that as IAV–host interaction research goes further, more host factors with important effects on IAV replication will be discovered, thus providing suitable targets for the research and development of anti-influenza drugs.

Our study identified for the first time that SNX16 could negatively regulate the replication of IAV: overexpression of SNX16 remarkably inhibited the replication of IAV, while knockdown of SNX16 by specific siRNA significantly promoted the replication of IAV. Interestingly, when the expression of SNX16 was detected, the transfection of SNX16-expressing constructs bearing different tags produced two obvious bands. Given the critical role of amino acid residue R144 in the function of SNX16, we determined whether the two-band expression pattern of SNX16 is related to the residue R144 by generating a SNX16 R144A mutant. We found that the introduction of the R144A mutation resulted in the disappearance of the upper SNX16 band, thus indicating that the two-band expression pattern of SNX16 is indeed determined by the amino acid residue R144. Furthermore, due to the R114A mutation, the distribution status of SNX16 was changed from condensed dots, which could be endosomes, to a diffuse state in the cytoplasm. Sequence analysis revealed that the amino acid residue R144 was highly conserved in SNX16 among different host species, suggesting that the R144 residue may play an important role in the function of SNX16.

When we examined the role of the R144 residue of SNX16 in the replication of IAV, we compared the growth titers of IAV in A549 cells overexpressing empty vector, Flag-SNX16, or Flag-SNX16^R144A^ mutant. We found that the R144A mutation partially reduced the inhibitory effect of SNX16 on the replication of IAV. These results indicate that the role of the R144 residue of SNX16 in restricting the replication of IAV may not be decisive but is indeed important. SNX16 is a membrane-binding protein and is localized in the endosomes and cell cortex. We speculate that the R144A mutation may have reduced the membrane-binding ability of SNX16, thereby leading to a changed distribution status and the reduced ability of restricting IAV replication of SNX16.

We finally explored the influence of SNX16 on the life cycle of IAV. During IAV infection, the accumulation of NP protein in the nucleus, a sign of the nuclear import of the vRNP complex, was largely delayed in SNX16-overexpressing HEK293T cells and was accelerated in SNX16-siRNA treated A549 cells, indicating that SNX16 negatively regulated the early stage of the IAV life cycle. It has been reported that during VSV infection, SNX16 acts as an effector of phosphatidylinositol 3-phosphate to regulate the export of the nucleocapsid of VSV from late endosomes, thereby inhibiting the replication of VSV (33). With respect to IAV, several early steps of its life cycle occur in endosomes. The localization of SNX16 in endosomes may confer it the ability to impair the viral transport, fusion, or uncoating process of IAV. Therefore, the role of SNX16 in suppressing the nuclear import of the vRNP complex of IAV is very likely an outcome of the restricted early steps in the endosomes.

## 5. Conclusions

Taken together, our study demonstrated that SNX16 is a novel restriction factor for the replication of IAV. SNX16 appears to inhibit the progression of the early stage of the IAV life cycle. Furthermore, a highly conserved amino acid residue, R144, largely determines the expression pattern and cellular distribution status of SNX16, and partially contributes to the inhibitory role of SNX16 in the replication of IAV. Given the critical anti-influenza function of SNX16, our study established that SNX16 could be a potential target for the development of antiviral drugs.

## Data Availability

Not applicable.
